# Clinical Events After Deferral of LAD Revascularization Following Physiological Coronary Assessment

**DOI:** 10.1016/j.jacc.2018.10.070

**Published:** 2019-02-05

**Authors:** Sayan Sen, Yousif Ahmad, Hakim-Moulay Dehbi, James P. Howard, Juan F. Iglesias, Rasha Al-Lamee, Ricardo Petraco, Sukhjinder Nijjer, Ravinay Bhindi, Sam Lehman, Darren Walters, James Sapontis, Luc Janssens, Christiaan J. Vrints, Ahmed Khashaba, Mika Laine, Eric Van Belle, Florian Krackhardt, Waldemar Bojara, Olaf Going, Tobias Härle, Ciro Indolfi, Giampaolo Niccoli, Flavio Ribichini, Nobuhiro Tanaka, Hiroyoshi Yokoi, Hiroaki Takashima, Yuetsu Kikuta, Andrejs Erglis, Hugo Vinhas, Pedro Canas Silva, Sérgio B. Baptista, Ali Alghamdi, Farrel Hellig, Bon-Kwon Koo, Chang-Wook Nam, Eun-Seok Shin, Joon-Hyung Doh, Salvatore Brugaletta, Eduardo Alegria-Barrero, Martijin Meuwissen, Jan J. Piek, Niels van Royen, Murat Sezer, Carlo Di Mario, Robert T. Gerber, Iqbal S. Malik, Andrew S.P. Sharp, Suneel Talwar, Kare Tang, Habib Samady, John Altman, Arnold H. Seto, Jasvindar Singh, Allen Jeremias, Hitoshi Matsuo, Rajesh K. Kharbanda, Manesh R. Patel, Patrick Serruys, Javier Escaned, Justin E. Davies

**Affiliations:** a Hammersmith Hospital, Imperial College London, Cancer Research UK, London, United Kingdom; b University College London Cancer Trials Centre, London, United Kingdom; c Lausanne University Hospital, Switzerland; d Royal North Shore Hospital, Sydney, New South Wales, Australia; e Flinders University, Adelaide, South Australia, Australia; f Prince Charles Hospital, Brisbane, Queensland, Australia; g MonashHeart and Monash University, Melbourne, Victoria, Australia; h Imelda Hospital, Bonheiden, Belgium; i Antwerp University Hospital, Antwerp, Belgium; j Ain Shams University, Cairo, Egypt; k Helsinki University Hospital, Helsinki, Finland; l INSERM Unité 1011, Lille, France; m Institut Coeur Poumon, Lille University Hospital, and Charite Campus Virchow Klinikum, Universitaetsmedizin, Berlin, Germany; n Gemeinschaftsklinikum Mittelrhein, Kemperhof Koblenz, Koblenz, Germany; o Sana Klinikum Lichtenberg, Lichtenberg, Germany; p Klinikum Oldenburg, European Medical School, Carl von Ossietzky University, Oldenburg, Germany; q University Magna Graecia, Catanzaro, Italy; r Catholic University of the Sacred Heart, Rome, Italy; s University Hospital Verona, Verona, Italy; t Tokyo Medical University, Tokyo, Japan; u Fukuoka Sannou Hospital, Fukuoka, Japan; v Aichi Medical University Hospital, Aichi, Japan; w Fukuyama Cardiovascular Hospital, Fukuyama, Japan; x Pauls Stradins Clinical University Hospital, Riga, Latvia; y Hospital Garcia de Horta, Lisbon, Portugal; z Hospital Santa Maria, Lisbon, Portugal; aa Hospital Prof. Doutor Fernando Fonseca, Amadora, Portugal; bb King Abdulaziz Medical City Cardiac Center, Riyadh, Saudi Arabia; cc Sunninghill Hospital, Johannesburg, Germany; dd Seoul National University Hospital, Seoul, South Korea; ee Keimyung University Dongsan Medical Center, Daegu, South Korea; ff Ulsan University Hospital, University of Ulsan College of Medicine, Ulsan, South Korea; gg Inje University Ilsan Paik Hospital, Daehwa-Dong, South Korea; hh Cardiovascular Institute, Hospital Clinic, Institut d’Investigacions Biomèdiques August Pi i Sunyer (IDIBAPS), Barcelona, Spain; ii Hospital Universitario de Torrejón and Universidad Francisco de Vitoria, Madrid, Spain; jj Amphia Hospital, Breda, Amsterdam, the Netherlands; kk AMC Heart Center, Academic Medical Center, Amsterdam, the Netherlands; ll VU University Medical Center, Amsterdam, the Netherlands; mm Istanbul University, Istanbul Faculty of Medicine, Istanbul, Turkey; nn Royal Brompton Hospital, and University of Florence, Florence, Italy; oo Conquest Hospital, St. Leonards-on-Sea, United Kingdom; pp Royal Devon and Exeter Hospital and University of Exeter, Exeter, United Kingdom; qq Royal Bournemouth General Hospital, Bournemouth, United Kingdom; rr Essex Cardiothoracic Centre, Basildon, and Anglia Ruskin University, Chelmsford, United Kingdom; ss Emory University, Atlanta, Georgia; tt Colorado Heart and Vascular, Lakewood, Colorado; uu Veterans Affairs Long Beach Healthcare System, Long Beach, California; vv Washington University School of Medicine, St. Louis, Missouri; ww Stony Brook University Medical Center, New York, New York; xx Gifu Heart Center, Gifu, Japan; yy John Radcliffe Hospital, Oxford University Hospitals Foundation Trust, Oxford, United Kingdom; zz Duke University, Durham, North Carolina; aaa Hospital Clinico San Carlos and Universidad Complutense de Madrid, Madrid, Spain

**Keywords:** coronary stenosis, fractional flow reserve, instantaneous wave-free ratio, FFR, fractional flow reserve, iFR, instantaneous wave-free ratio, LAD, left anterior descending, MACE, major adverse cardiac events

## Abstract

**Background:**

Physicians are not always comfortable deferring treatment of a stenosis in the left anterior descending (LAD) artery because of the perception that there is a high risk of major adverse cardiac events (MACE). The authors describe, using the DEFINE-FLAIR (Functional Lesion Assessment of Intermediate Stenosis to Guide Revascularisation) trial, MACE rates when LAD lesions are deferred, guided by physiological assessment using fractional flow reserve (FFR) or the instantaneous wave-free ratio (iFR).

**Objectives:**

The purpose of this study was to establish the safety of deferring treatment in the LAD using FFR or iFR within the DEFINE-FLAIR trial.

**Methods:**

MACE rates at 1 year were compared between groups (iFR and FFR) in patients whose physiological assessment led to LAD lesions being deferred. MACE was defined as a composite of cardiovascular death, myocardial infarction (MI), and unplanned revascularization at 1 year. Patients, and staff performing follow-up, were blinded to whether the decision was made with FFR or iFR. Outcomes were adjusted for age and sex.

**Results:**

A total of 872 patients had lesions deferred in the LAD (421 guided by FFR, 451 guided by iFR). The event rate with iFR was significantly lower than with FFR (2.44% vs. 5.26%; adjusted HR: 0.46; 95% confidence interval [CI]: 0.22 to 0.95; p = 0.04). This was driven by significantly lower unplanned revascularization with iFR and numerically lower MI (unplanned revascularization: 2.22% iFR vs. 4.99% FFR; adjusted HR: 0.44; 95% CI: 0.21 to 0.93; p = 0.03; MI: 0.44% iFR vs. 2.14% FFR; adjusted HR: 0.23; 95% CI: 0.05 to 1.07; p = 0.06).

**Conclusions:**

iFR-guided deferral appears to be safe for patients with LAD lesions. Patients in whom iFR-guided deferral was performed had statistically significantly lower event rates than those with FFR-guided deferral.

The instantaneous wave-free ratio (iFR) (1) is an index of stenosis severity that has been demonstrated to be noninferior to fractional flow reserve (FFR) when guiding coronary revascularization 2, 3. iFR does not require adenosine and can be performed in a significantly shorter time than FFR (2). In the DEFINE-FLAIR (Functional Lesion Assessment of Intermediate Stenosis to Guide Revascularisation) study, iFR was found to defer revascularization in a significantly higher proportion of patients (2). Although iFR was found to be noninferior to FFR on a population basis, the applicability of this result to individual patients depends on understanding how these 2 indexes guide revascularization in different subsets of patients.

Leaving a lesion in the left anterior descending (LAD) artery unstented because of physiological guidance can cause physicians to be concerned about the potential for subsequent events, because the LAD often supplies a large territory of myocardium (4). It has been suggested that because of the relatively large area of myocardium at risk, only hyperemia-based indexes can be trusted for decision making in the LAD 5, 6.

In this analysis, we present 1-year major adverse cardiac events (MACE) rates of patients who have physiologically-guided deferral of LAD revascularization in the iFR and FFR arms of the DEFINE-FLAIR trial.

## Methods

### Study population

**Figure 1:**
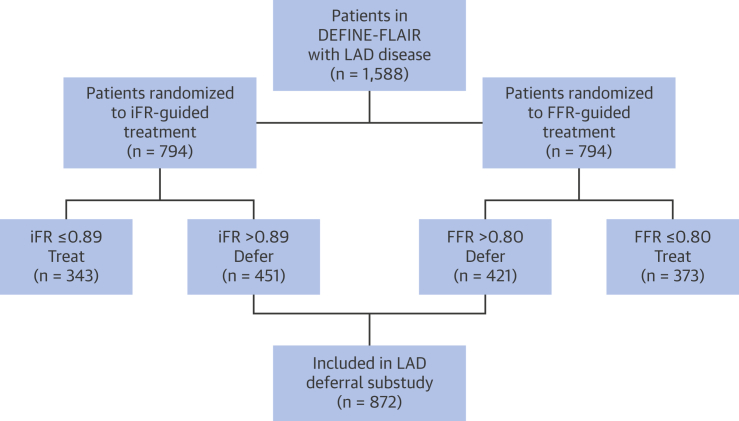
Flow Chart Outlining Patient Selection Patients were included from the DEFINE-FLAIR trial. This analysis was focused on patients who had lesions within their LAD, and who then went on to be deferred on the basis of intracoronary physiology (either iFR or FFR). The total number of patients included in the LAD deferred analysis was 872. DEFINE-FLAIR = Functional Lesion Assessment of Intermediate Stenosis to Guide Revascularisation; FFR = fractional flow reserve; iFR = instantaneous wave-free ratio; LAD = left anterior descending.

All patients in the DEFINE-FLAIR study who had treatment of their LAD stenosis deferred, based on an iFR >0.89 or an FFR >0.80, were included in this study (Figure 1). The inclusion and exclusion criteria for the DEFINE-FLAIR study has been described in detail elsewhere (2). To summarize, DEFINE-FLAIR included all patients with angiographically-determined moderate stenoses. Notable exclusions included those with left main disease, prior coronary artery bypass surgery, or significant valve disease.

### Study protocol

#### Cardiac catheterization

Coronary angiography was performed via the transradial or transfemoral route at the operator’s discretion. Before physiological measurements were made, intracoronary nitrates were administered to control vasomotor tone. FFR and iFR measurements were then performed in all appropriate vessels in the routine manner using a coronary pressure guidewire. Pre-specified treatment cutpoints were an FFR of 0.80 and an iFR of 0.89. Revascularization was performed when the physiological value was equal to or lower than these pre-specified thresholds, and revascularization was deferred when it was above these thresholds.

LAD territory patients are defined as patients undergoing physiological assessment, which included LAD assessment in which the LAD was deferred based on the iFR or FFR measurement (Figure 1). Non-LAD territory patients are defined as patients undergoing physiological assessment that did not include LAD assessment in which intervention was deferred in at least 1 vessel (either Cx or RCA) based on the iFR or FFR measurement (Figure 1). MACE was defined as the composite of cardiovascular death, myocardial infarction, and unplanned revascularization. This differs slightly from the DEFINE-FLAIR trial, where MACE was defined as the composite of all-cause mortality, myocardial infarction, and unplanned revascularization. Both definitions of MACE will be reported in this study.

MI was classified as either spontaneous or periprocedural and as either ST-segment elevation or non–ST-segment elevation MI. Spontaneous MI was defined as an event after the first 48 h post–percutaneous coronary intervention (PCI) or 7 days following coronary artery bypass graft, unrelated to the procedure. MI was defined by a typical rise and gradual fall (troponin) or more rapid rise and fall (creatine kinase-MB) of biochemical markers of myocardial necrosis with at least 1 of the following: ischemic symptoms; development of new pathological Q waves on the ECG; and/or ECG changes indicative of ischemia. Periprocedural MI was considered an event within the first 48 h after PCI and within 7 days following coronary artery bypass graft. Revascularization was considered to be unplanned when it was not the index procedure and was not identified at the time of the index procedure as a staged procedure to occur within 60 days. Additionally, unplanned revascularization required symptoms consistent with ischemia. All events were independently adjudicated.

### Statistical analysis

The objective of this study was to compare event rates between physiology techniques (iFR vs. FFR) in patients for whom revascularization was deferred, separately in LAD territory patients and non-LAD territory patients.

Baseline and procedural characteristics of patients were analyzed in the following manner. Categorical and binary variables were compared between groups using chi-square tests. Continuous variables were compared using Student’s *t*-test, or Wilcoxon signed-rank test in case of non-normal distributions.

For MACE and its components, a time-to-event analysis was performed by Cox survival modeling. Participants who withdrew from the study before reaching 1 year of follow-up and who were event-free at their last visit were censored at their time of last visit. Testing of validity of proportional hazard assumption was done using Schoenfeld residuals. There were no signs of violations of proportional hazards assumption.

Results are reported using hazard ratios (HRs), 95% 2-sided confidence intervals (CIs) and cumulative hazard curves. Analyses were performed in an unadjusted manner. In addition, adjustment for age and sex was performed. Indeed, despite randomization at the trial level, sex was found to be imbalanced between iFR and FFR groups in this study of deferred patients. Moreover, iFR patients were slightly younger than FFR patients, although this difference did not reach statistical significance. Results are presented as adjusted for age and sex in text, and both as unadjusted and adjusted in the tables.

## Results

### LAD artery

The baseline characteristics of patients who had revascularization deferred in their LAD are shown in Table 1. Patients in the iFR group were more likely to be male (75.4% iFR vs. 68.9% FFR; p = 0.03). There were no other statistical differences in baseline characteristics between the iFR and FFR arms in patients with LAD territory disease who had revascularization deferred on the basis of intracoronary physiology. The majority of patients were classified with stable disease. A total of 80 patients (17.7%) in the iFR group and 75 patients (17.8%) in the FFR group had an acute coronary syndrome. There was no significant difference between iFR and FFR groups in proportions of patients taking aspirin (p = 0.65), statins (p = 0.78), beta-blockers (p = 0.39), angiotensin-converting enzyme inhibitors (p = 0.21), angiotensin receptor blockers (p = 0.40), and calcium channel antagonists (p = 0.66). There were no significant differences in medical therapy at 30 days and 1 year. For treated patients, all were treated with drug-eluting stents.

**Table 1:** Baseline Characteristics of Deferred Patients

	LAD Artery			Non-LAD Artery		
	iFR (n = 451)	FFR (n = 421)	p Value	iFR (n = 343)	FFR (n = 327)	p Value
Age, yrs	64.8 ± 11.0	66.1 ± 10.4	0.09	64.9 ± 11.4	65.5 ± 10.5	0.48
Male	340 (75.4)	290 (68.9)	0.03	267 (77.8)	232 (70.9)	0.04
Diabetes	121 (26.8)	117 (27.8)	0.4	97 (28.3)	112 (34.3)	0.21
Hypertension	311 (69.0)	299 (71.0)	0.77	238 (69.4)	241 (73.7)	0.45
Hyperlipidemia	281 (62.3)	268 (63.7)	0.82	223 (65.0)	211 (64.5)	0.46
Acute coronary syndrome	80 (17.7)	75 (17.8)	0.42	70 (20.4)	65 (19.9)	0.87
Previous myocardial infarction	111 (24.6)	97 (23.0)	0.8	108 (31.5)	107 (32.7)	0.94
Previous PCI	142 (31.5)	143 (34.0)	0.42	162 (47.2)	157 (48.0)	0.8

Values are mean ± SD or n (%).

FFR = fractional flow reserve; iFR = instantaneous wave-free ratio; LAD = left anterior descending; PCI = percutaneous coronary intervention.

A total of 1,588 patients in DEFINE-FLAIR had physiological assessment in the LAD territory in total (794 patients in each arm). The proportion of patients with >1 evaluated stenosis was similar between iFR and FFR at 31.0% (n = 1,106) and 32.9% (n = 1,129), respectively (p = 0.42). The mean iFR value was 0.89 ± 0.09, and the mean FFR value 0.82 ± 0.09 in the LAD territory. When using iFR or FFR to guide revascularization in the LAD territory, the 1-year MACE rate was 4.03% for iFR and 5.54% for FFR in all patients, both treated and deferred (adjusted HR: 0.66; 95% CI: 0.40 to 1.08; p = 0.1).

The proportion of patients with 0, 1, or >1 hemodynamically significant stenosis was 55.3%, 42.2%, and 2.5% in iFR, and 51.3%, 41.4%, and 7.2% in FFR (p < 0.01). There were 376 hemodynamically significant stenoses in the iFR arm and 452 in the FFR arm. Stent length and diameter was equivalent between iFR and FFR groups. The procedural characteristics for patients in whom revascularization in the LAD was deferred on the basis of physiology is summarized in Table 2.

**Table 2:** Procedural Characteristics of Deferred Patients

	LAD Artery			Non-LAD Artery		
	iFR (n = 451)	FFR (n = 421)	p Value	iFR (n = 343)	FFR (n = 327)	p Value
Radial access	327 (72.5)	308 (73.2)	0.97	246 (71.7)	238 (72.8)	0.76
Total number of evaluated lesions	634	574	—	378	357	0.76
Mean iFR/FFR value	0.94 ± 0.03	0.87 ± 0.04	—	0.97 ± 0.03	0.91 ± 0.05	—

Values are n (%), n, or mean ± SD.

Abbreviations as in Table 1.

The mean iFR was 0.94 ± 0.03 versus mean FFR value 0.87 ± 0.04 in deferred stenoses. Among the 872 patients with deferred LAD lesions, there was no difference in lesion location. A total of 40% of patients in the FFR arm had proximal LAD lesions versus 37% with iFR (p = 0.23); 55% of patients in the FFR arm had mid LAD lesions versus 57% with iFR (p = 0.50); and 2% of patients in the FFR arm had distal LAD lesions versus 4% with iFR (p = 0.13).

**Figure 2:**
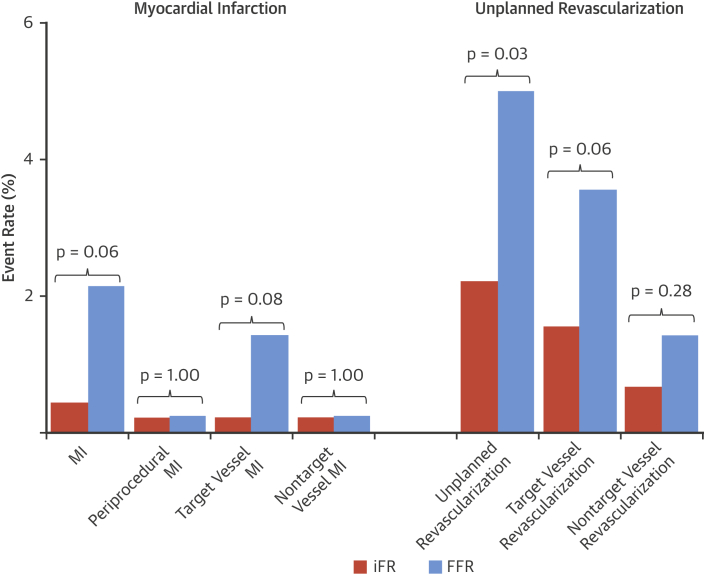
Summary of Clinical Events in LAD-Deferred Patients Bar charts outlining clinical events in patients with LAD stenoses deferred on the basis of intracoronary physiology. The **orange bars** denote patients whose treatment was guided by iFR, and the **blue bars** denote patients whose treatment was guided by FFR. iFR-guided deferral was associated with significantly lower rates of unplanned revascularization (**right**, p = 0.03). This was driven by numerically greater rates of target vessel revascularization with FFR (p = 0.06). iFR-guided deferral was associated with numerically lower rates of myocardial infarction (MI) (**left**, p = 0.06). This was driven by numerically greater rates of target vessel MI with FFR (p = 0.08). There was no difference in periprocedural MI (p = 1.00). Abbreviations as in Figure 1.

iFR-based deferral of LAD stenoses was associated with a MACE rate of 2.44% at 1 year compared with a MACE rate of 5.46% with FFR (adjusted HR: 0.46; 95% CI: 0.22 to 0.95; p = 0.04) (Figure 2, Central Illustration). This difference was driven by numerically greater unplanned revascularization and myocardial infarction.

**Central Illustration:**
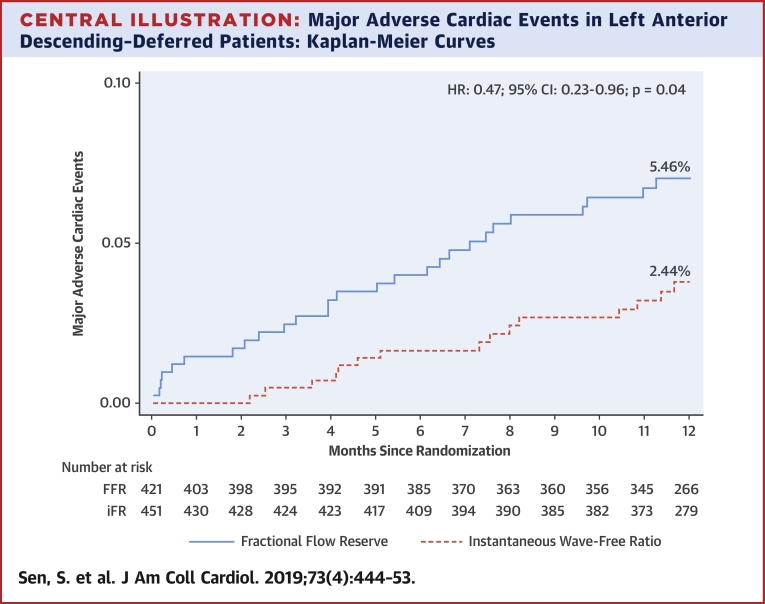
Major Adverse Cardiac Events in Left Anterior Descending–Deferred Patients: Kaplan-Meier Curves This figure outlines the primary endpoint in patients with left anterior descending stenoses who were deferred according intracoronary physiology. Major adverse cardiac events were defined as the composite of cardiovascular death, myocardial infarction, and unplanned revascularization. The **solid blue line** denotes the fractional flow reserve arm, and the **dashed orange line** denotes the instantaneous wave-free ratio arm. Instantaneous wave-free ratio–guided deferral was associated with a significantly lower major adverse cardiac events rate (adjusted hazard ratio: 0.46; 95% confidence interval: 0.22 to 0.95; p = 0.04).

Unplanned revascularization occurred in 10 patients (2.22%) in the iFR-deferred group and 21 patients (4.99%) in the FFR-deferred group (adjusted HR 0.44; 95% CI: 0.21 to 0.93; p = 0.03) (Table 3, Figure 2). The target vessel was revascularized in 7 patients (1.55%) of the iFR group compared with 15 patients (3.56%) of the FFR group (adjusted HR: 0.42; 95% CI: 0.17 to 1.04; p = 0.06).

**Table 3:** Clinical Outcomes in LAD-Deferred Patients

	Unadjusted				Adjusted	
	iFR Group (n = 451)	FFR Group (n = 421)	Hazard Ratio (95% CI)	p Value	Hazard Ratio (95% CI)	p Value
MACE (cardiovascular death, MI, unplanned revascularization)	11 (2.44)	23 (5.46)	0.47 (0.23–0.96)	0.04∗	0.46 (0.22–0.95)	0.04∗
Cardiovascular death	0 (0.00)	1 (0.24)				
MACE (all-cause death, MI, unplanned revascularization)	15 (3.33)	27 (6.41)	0.54 (0.29–1.02)	0.06	0.53 (0.28–1.01)	0.05
All-cause death	4 (0.89)	5 (1.19)	0.76 (0.21–2.84)	0.69	0.77 (0.21–2.86)	0.69
MI	2 (0.44)	9 (2.14)	0.24 (0.05–1.11)	0.07	0.23 (0.05–1.07)	0.06
Target vessel MI	1 (0.22)	6 (1.43)	0.16 (0.02–1.30)	0.09	0.15 (0.02–1.23)	0.08
Nontarget vessel MI	0 (0.00)	2 (0.48)				
Periprocedural MI	1 (0.22)	1 (0.24)	1.00 (1.00–1.00)	1.00	1.00 (1.00–1.00)	1.00
Unplanned revascularization	10 (2.22)	21 (4.99)	0.45 (0.21–0.95)	0.04∗	0.44 (0.21–0.93)	0.03∗
TVR	7 (1.55)	15 (3.56)	0.44 (0.18–1.07)	0.07	0.42 (0.17–1.04)	0.06
Non-TVR	3 (0.67)	6 (1.43)	0.47 (0.12–1.88)	0.29	0.47 (0.12–1.88)	0.28

Values are n (%) unless otherwise indicated.

CI = confidence interval; MACE = major adverse cardiac events; MI = myocardial infarction; TVR = target vessel revascularization; other abbreviations as in Table 1.

∗ Statistically significant p values.

Myocardial infarction occurred in 2 patients after iFR-guided LAD deferral at 1 year compared with 9 patients after FFR-guided deferral (0.44% iFR vs. 2.14% FFR; adjusted HR: 0.23; 95% CI: 0.05 to 1.07; p = 0.06) (Table 3, Figure 2). Target vessel MI occurred in 1 patient in iFR compared with 6 patients with FFR (0.22% vs. 1.43%; adjusted HR: 0.15; 95% CI: 0.02 to 1.23; p = 0.08) (Figure 2).

### Non-LAD territory

The baseline characteristics of patients who had revascularization deferred in the non-LAD territories are shown in Table 1. Patients in the iFR group were more likely to be male (77.8% iFR vs. 70.9% FFR; p = 0.04). There were no other statistical differences in baseline characteristics between the iFR and FFR arms in patients with non-LAD territory disease who had revascularization deferred on the basis of intracoronary physiology (Table 1). The majority of patients were classified with stable disease. A total of 70 patients (20.4%) in the iFR group and 65 patients (19.9%) in the FFR group had an acute coronary syndrome.

A total of 834 patients had physiological assessment in the non-LAD territory (409 iFR, 425 FFR). There were 457 stenosis in the iFR arm and 470 stenoses in the FFR arm. The proportion of patients with >1 evaluated vessel was similar between iFR and FFR, at 11.2% and 9.4%, respectively (p = 0.38). The mean iFR value was 0.95 ± 0.10 and the mean FFR value 0.87 ± 0.10 in the non-LAD territory. The event rate in the overall population (both treated and deferred) with non-LAD disease was 7.58% with iFR and 6.35% with FFR (HR: 1.27; 95% CI: 0.73 to 2.21; p = 0.4).

The proportion of patients with 0, 1, or >1 hemodynamically significant stenosis was 83.9%, 15.2%, and 1.0% in iFR, and 76.9%, 21.9%, and 1.2% in FFR (p = 0.04). The number of significant stenoses was significantly lower in the iFR arm (70 vs. 104; p = 0.01). Stent length and diameter was equivalent between iFR and FFR groups; however, more stents were placed in the FFR arm. The procedural characteristics for patients in whom revascularization in the non-LAD territories was deferred on the basis of physiology is summarized in Table 2.

**Figure 3:**
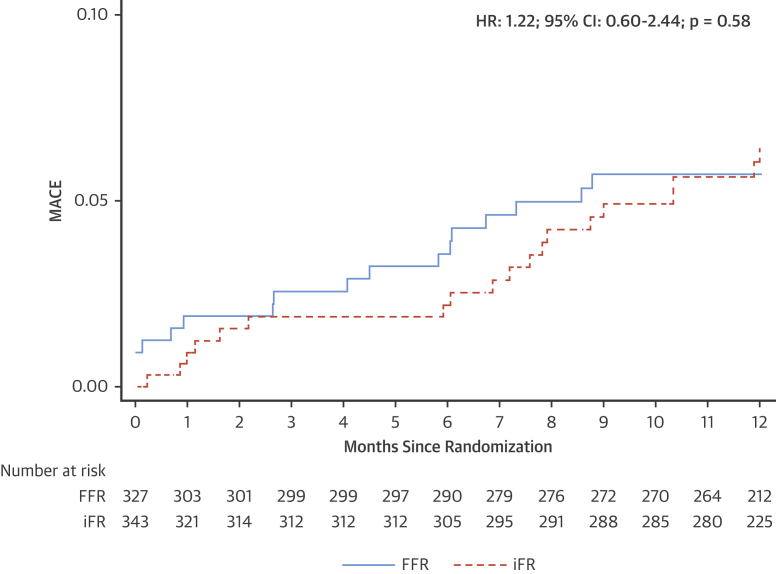
Kaplan-Meier for MACE in Non-LAD Patients Primary endpoint in patients with non-LAD stenoses who were deferred according to intracoronary physiology. MACE was defined as the composite of cardiovascular death, myocardial infarction, and unplanned revascularization. The **solid blue line** denotes the FFR arm, and the **dashed orange line** denotes the iFR arm. There was no difference in the MACE rate between iFR- and FFR-guided deferral (adjusted hazard ratio: 1.18; 95% confidence interval: 0.59 to 2.38; p = 0.63). Abbreviations as in Figure 1.

The mean iFR was 0.97 ± 0.03 versus mean FFR value 0.91 ± 0.05 in deferred stenoses. iFR-based deferral of non-LAD stenoses was associated with a MACE rate of 5.25% at 1 year compared with a MACE rate of 5.20% with FFR (adjusted HR: 1.18; 95% CI: 0.59 to 2.38; p = 0.63) (Figure 3).

Unplanned revascularization occurred in 15 patients in the iFR-deferred group and 16 patients in the FFR-deferred group (4.37% iFR vs. 4.89% FFR; adjusted HR: 0.98; 95% CI: 0.47 to 2.04; p = 0.97) (Table 4). The target vessel was revascularized in 5 patients of the iFR group compared with 10 patients of the FFR group (1.46% iFR vs. 3.06% FFR; adjusted HR: 0.52; 95% CI: 0.17 to 1.55; p = 0.24).

**Table 4:** Clinical Outcomes in Non–LAD-Deferred Patients

	Unadjusted				Adjusted	
	iFR Group (n = 343)	FFR Group (n = 327)	Hazard Ratio (95% CI)	p Value	Hazard Ratio (95% CI)	p Value
MACE (cardiovascular death, MI, unplanned revascularization)	18 (5.25)	17 (5.20)	1.22 (0.60–2.44)	0.58	1.18 (0.59–2.38)	0.63
Cardiovascular death	3 (0.87)	0 (0.00)				
MACE (all-cause death, MI, unplanned revascularization)	20 (5.83)	21 (6.42)	1.05 (0.56–1.99)	0.88	1.04 (0.55–1.97)	0.9
All-cause death	5 (1.46)	4 (1.22)	1.20 (0.32–4.48)	0.78	1.28 (0.34–4.76)	0.72
MI	5 (1.46)	6 (1.83)	1.20 (0.32–4.45)	0.79	1.09 (0.29–4.08)	0.89
Target vessel MI	2 (0.58)	2 (0.61)	0.96 (0.14–6.83)	0.97	0.87 (0.12–6.15)	0.88
Non-target vessel MI	3 (0.87)	2 (0.61)	1.43 (0.24–8.54)	0.7	1.32 (0.22–7.92)	0.76
Periprocedural MI	0 (0.00)	2 (0.61)				
Unplanned revascularization	15 (4.37)	16 (4.89)	1.02 (0.49–2.10)	0.97	0.98 (0.47–2.04)	0.97
TVR	5 (1.46)	10 (3.06)	0.53 (0.18–1.57)	0.25	0.52 (0.17–1.55)	0.24
Non-TVR	10 (2.92)	6 (1.83)	1.90 (0.65–5.55)	0.24	1.80 (0.62–5.29)	0.28

Values are n (%) unless otherwise indicated.

Myocardial infarction occurred in 5 patients after iFR-guided non-LAD deferral at 1 year compared with 6 patients after FFR-guided deferral (1.46% iFR vs. 1.83% FFR; adjusted HR: 1.09; 95% CI: 0.29 to 4.08; p = 0.89) (Table 4). Target vessel MI occurred in 2 patients in iFR and 2 patients with FFR (0.58% vs. 0.61%; adjusted HR: 0.87; 95% CI: 0.12 to 6.15; p = 0.88).

## Discussion

Physiologically-guided deferral of revascularization in the LAD is not associated with an unacceptably high MACE rate. The outcomes of patients having this deferral guided by iFR rather than by FFR are no worse, and may be better.

### Deferral of intervention based on FFR

The principle of physiological guidance of revascularization is to identify lesions in which deferral is likely to be safe (7). Among angiographically moderate lesions, FFR has been shown to successfully identify lesions that can safely be managed conservatively (8). This manifests as a reduction in unplanned revascularization and MI with FFR-guided revascularization when compared with angiography-guided revascularization. This ability to reduce MI and unplanned revascularization led to a paradigm shift away from angiography-guided intervention to FFR-guided intervention and was pivotal in the adoption of FFR into guidelines 9, 10.

The ability of FFR to discern the risk of stenoses has not been compared with other indexes in a randomized and prospective fashion until DEFINE-FLAIR. Furthermore, DEFINE-FLAIR was the first study to include a predominance of stenoses most commonly represented in clinical practice. When FFR has been previously studied in this distribution of patients, there has been some concern as to its safety and, therefore, utility in guiding revascularization (11).

Within DEFINE-FLAIR, in non-LAD lesions, FFR-guided deferral had similar outcomes to iFR-guided deferral. For LAD lesions, there was a trend toward better outcomes in those with iFR-guided deferral than those with FFR-guided deferral. The difference in the composite endpoint was driven by statistically higher unplanned revascularization and numerically higher myocardial infarction in the LAD in the FFR arm.

### Potential challenges for hyperemia-dependent indexes in the LAD

When flow measurements are available, previous work has indicated that coronary flow reserve (CFR), the extent of hyperemic flow during adenosine administration, is the most powerful predictor of events 12, 13, 14. Patients with impaired CFR have a worse prognosis. Recent studies have demonstrated that patients with abnormal CFR but normal FFR are prone to higher adverse event rates (15). The discordance of CFR to FFR can be as high as 40% (16). This may, therefore, lead to the possibility that a proportion of patients in the FFR arm of this study had abnormal CFR values, and this predisposed them to higher event rates in the FFR arm.

The same could also apply to iFR; however, the agreement between iFR and CFR has been demonstrated to be significantly closer than that of FFR and CFR (17). Therefore, the proportion of patients in whom iFR is normal and CFR abnormal is lower, possibly explaining the lower event rate in the iFR-deferred patients (15).

### iFR-based deferral

Physicians are correct to question the safety of any technique proposed as an alternative to one that is accepted as safe. The LAD has been highlighted as a territory in which reliance on nonhyperemic indexes may be particularly dangerous 5, 6. This is because the LAD supplies a large amount of myocardium, and any index of stenosis severity will need to also reflect the amount of myocardium subtended by the vessel. It has been assumed that the amount of myocardium can only be appreciated during hyperemia.

This analysis suggests that, for patients having LAD lesions deferred based on physiology, the event rate is not higher than that of patients in which non-LAD lesions are deferred. Furthermore, there is no sign of increased risk if guided by iFR compared with those guided by FFR. This suggests that hyperemia is not required to safely defer lesions in the left anterior descending artery.

### Study limitations

This is a post hoc analysis of DEFINE-FLAIR regarding the safety of deferral of PCI using nonhyperemic physiology, particularly in the LAD 5, 6. This is not itself a randomized controlled trial. However, it benefits from the blinding provided by the DEFINE FLAIR trial, which ensures that the patients and their clinical care staff were unaware of allocation arm so that decisions to revascularize were unbiased. Additionally, the endpoint adjudication committee was blinded to allocation arm.

Our findings should be considered hypothesis-generating, and further research in the field is necessary. Nevertheless, our results confirm there is no safety hazard for using iFR to defer lesions in the LAD. There were only 872 patients with LAD deferral. However, this is not unsatisfactory by comparison to the dataset upon which the safety of FFR deferral was established, because it is greater than the total number of patients undergoing physiological deferral in DEFER (Fractional Flow Reserve to Determine the Appropriateness of Angioplasty in Moderate Coronary Stenosis) (n = 91 patients) and FAME (Fractional Flow Reserve versus Angiography for Guiding Percutaneous Coronary Intervention) (n ≤509 patients).

## Conclusions

iFR-guided deferral appears to be safe for patients with LAD lesions. Patients in whom iFR-guided deferral was performed had statistically significantly lower event rates than those with FFR-guided deferral. Further studies would be useful to explore these findings in more detail.

Perspectives **COMPETENCY IN PATIENT CARE AND PROCEDURAL SKILLS:** Deferral of LAD revascularization based on assessment of coronary physiology by iFR was associated with lower adverse cardiac event rates than when decisions were guided by measurement of FFR.

**TRANSLATIONAL OUTLOOK:** Future studies should compare available methods of evaluating the physiological impact of stenotic coronary artery lesions on clinical outcomes in patients with other anatomical patterns of disease.
